# Fabrication of Maghemite Nanoparticles with High Surface Area

**DOI:** 10.3390/nano9071004

**Published:** 2019-07-12

**Authors:** Yulia Trushkina, Cheuk-Wai Tai, German Salazar-Alvarez

**Affiliations:** Department of Materials and Environmental Chemistry, Arrhenius Laboratory, Stockholm University, SE-10691 Stockholm, Sweden

**Keywords:** porous materials, iron oxide, nanostructures, transformation, characterization

## Abstract

Maghemite nanoparticles with high surface area were obtained from the dehydroxylation of lepidocrocite prismatic nanoparticles. The synthesis pathway from the precursor to the porous maghemite nanoparticles is inexpensive, simple and gives high surface area values for both lepidocrocite and maghemite. The obtained maghemite nanoparticles contained intraparticle and interparticle pores with a surface area ca. 30 × 10^3^ m^2^/mol, with pore volumes in the order of 70 cm^3^/mol. Both the surface area and pore volume depended on the heating rate and annealing temperature, with the highest value near the transformation temperature (180–250 °C). Following the transformation, in situ X-ray diffraction (XRD) allowed us to observe the temporal decoupling of the decomposition of lepidocrocite and the growth of maghemite. The combination of high-angle annular dark-field imaging using scanning transmission electron microscopy (HAADF-STEM) and surface adsorption isotherms is a powerful approach for the characterization of nanomaterials with high surface area and porosity.

## 1. Introduction

Iron oxide nanoparticles are available nowadays in a variety of shapes and are technologically important components widely used for the production of, e.g., magnetic materials [1,2,3], color pigments [4,5], catalysts [6], sorbents [7,8], for biomedical applications and drug delivery [3,9,10,11,12], as well as being key components in the Martial soil [13]. Very recently, iron oxides have become very popular as electrodes for lithium-ion, sodium-ion and alkaline-ion batteries [14,15,16,17,18]. In terms of adsorption and storage purposes [19,20,21,22], parameters such as surface area, pore size, and porosity are of great importance for mass transport [23].

Synthetic lepidocrocite nanoparticles offer an opportunity to study pore formation in iron (oxy)hydroxides. During heating, lepidocrocite (Lp) transforms to maghemite (Mh) between 200 and 280 °C under air at standard temperature and pressure (STP) conditions or at 120–268 °C under vacuum with typical heating rates of 0.5–10 °C/min [24,25,26,27,28,29,30,31]. The weight loss is ~12% for both conditions, as expected for the dexydroxylation reaction 2FeOOH → Fe_2_O_3_ + H_2_O [25,26,32]. During transformation, random nucleation of maghemite occurs, producing perfectly oriented polycrystalline agglomerates [25,28,33]. Giovanoli and Brutsch [25] suggested that lepidocrocite layers collapse in such a way that more edge and corner sharing O^2−^ ions become available to form H_2_O on the one hand and form a ccp of O^2−^ on the other hand. Both processes involve a strain in the lattice whereby pores form to at least partly compensate for the lattice distortion. A common feature of the dehydroxylation of all iron (oxy)hydroxides is the initial development of microporosity due to the expulsion of water [4,34,35]. Prolongation of the heating leads to the coalescence of micropores to mesopores and macropores. Pore formation is accompanied by a rise in sample surface area, and the degree of porosity depends on the heating treatment conditions.

Naono and Fujiwara [34,35] reported that during the thermal treatment of α-FeOOH goethite and β-FeOOH akaganeite, nanoparticles obtained micro- and mesopores with a slit shape and a width of 0.8–3 nm. Cornell and Schwertmann [4] reported that surface area values of synthetic lepidocrocite range from ≈1000 to 23000 m^2^/mol, with low area values (≈3000 m^2^/mol) for samples obtained from Fe^2+^ systems. For maghemite obtained from Lp, the surface area values range from 8000 to 20000 m^2^/mol [4,25]. Naono and Nakai [28] reported specific surface area values (nitrogen adsorption) from ≈2000 m^2^/mol for room temperature Lp acicular nanoparticles to 16,000 m^2^/mol for an Mh sample heated to 200 °C. They also reported 2–3 nm pore sizes (width), as determined by transmission electron microscopy (TEM), and 0.9 nm from t-plot data for slit-shape pores formed during transformation.

In this work, we have studied the transformation of lepidocrocite nanoprisms into porous maghemite nanoprisms. The structural transformation was followed by in situ X-ray diffraction, which points to a time delay between the decomposition of lepidocrocite and the nucleation of maghemite. Nitrogen adsorption measurements show a large increase in the mesopore volume during the transformation, with a broad size of mesopores and negligible microporosity. The obtained materials have a large surface area ca. 30 × 10^3^ m^2^/mol, with pore volumes about 70 cm^3^/mol. Transmission electron microscopy images indicate a large number of intraparticular small pores/roughness in the range 2–3 nm and interparticular pores with a broad size distribution. The fabrication of highly porous particles can benefit novel electrode materials where the detailed information regarding the porosity of the samples is crucial for the understanding of their properties.

## 2. Materials and Methods

Synthesis and Transformation. Prismatic shaped lepidocrocite nanoparticles were prepared by precipitation and oxidation of iron (II) chloride at room temperature as described in Kozin et al. [36]. Iron (II) chloride tetrahydrate (98%, Sigma-Aldrich, Stockholm, Sweden) and sodium hydroxide (99%, VMR Chemicals, Stockholm, Sweden) were used as received without further purification. For the preparation of a stock solution, pre-boiled deionized MilliQ water was used. Nanoparticles were synthesized from a 500 mL solution of 0.06 M FeCl_2_. Prior to the synthesis, the solution was filtered to remove adventitious minerals, such as akaganeite, and possible insoluble contaminants. The pH of the solution was thereafter adjusted to 7 with 1 M NaOH. Purified air was flushed through the resulting solution to induce the formation of nanoparticles by oxidation of the ferrous iron. The pH of the suspension was kept at 7 throughout the course of the synthesis by drop-wise addition of NaOH. A resulting dark greenish-grey (green rust) precipitate formed at the early stages of oxidation that ultimately turned into bright orange crystals within 3 h. The resulting orange solution was washed several times with hydrochloric acid solution at pH = 3, followed by dialysis in deionized water for 24 h. In order to obtain powder specimens, portions of resulting stock suspension were dried in an oven at 60 °C for 24 h.

In order to transform lepidocrocite to maghemite, the powder specimen of lepidocrocite was heated in a furnace under air at heating rates of 0.1–10 °C/min.

In Situ Powder X-ray Diffraction (iXRD). Powder specimens were ground in agate mortar prior to measurements. Aluminum pans (PerkinElmer Inc., Farsta, Sweden) were used as sample holders in all powder X-ray diffraction measurements and mounted in a computer-controlled Linkam TS1500 stage (Linkam Scientific Instruments, Tadworth, UK). iXRD patterns were acquired in transmission mode with an Xcalibur-III Single Crystal diffractometer (Oxford Diffraction Limited, Oxfordshire, UK) using Mo radiation. Two frames per each of four detector positions between ± 36° were collected at a sample to detector distance of 100 mm with a Sapphire 3 charged coupled device (CCD) detector (2040 × 2040 pixels, pixel size = 30.2 × 30.2 µm^2^) using an acquisition time of 60 s/frame. The frames covered a range of 0°–55° 2θ. The obtained patterns were analyzed using the program Origin.

Transmission Electron Microscopy (TEM). The specimens were prepared by depositing a drop of a dilute aqueous dispersion of nanoparticles on carbon-coated copper grids dried under atmospheric conditions. The images were collected with a JEOL JEM-2100F (JEOL Ltd., Japan) microscope with a Schottky-type field emission gun operated at 200 kV (Cs = 0.5 mm, Cc = 1.1 mm) equipped with a Gatan Ultrascan 1000 camera (2048 × 2048 pixel and pixel size = 14 × 14 μm^2^) and JEOL annular dark-field (ADF) detectors (for high-angle annular dark-field imaging using scanning transmission electron microscopy (HAADF-STEM) imaging). The camera length used in HAADF-STEM was either 8 or 10 cm.

**Figure 1:**
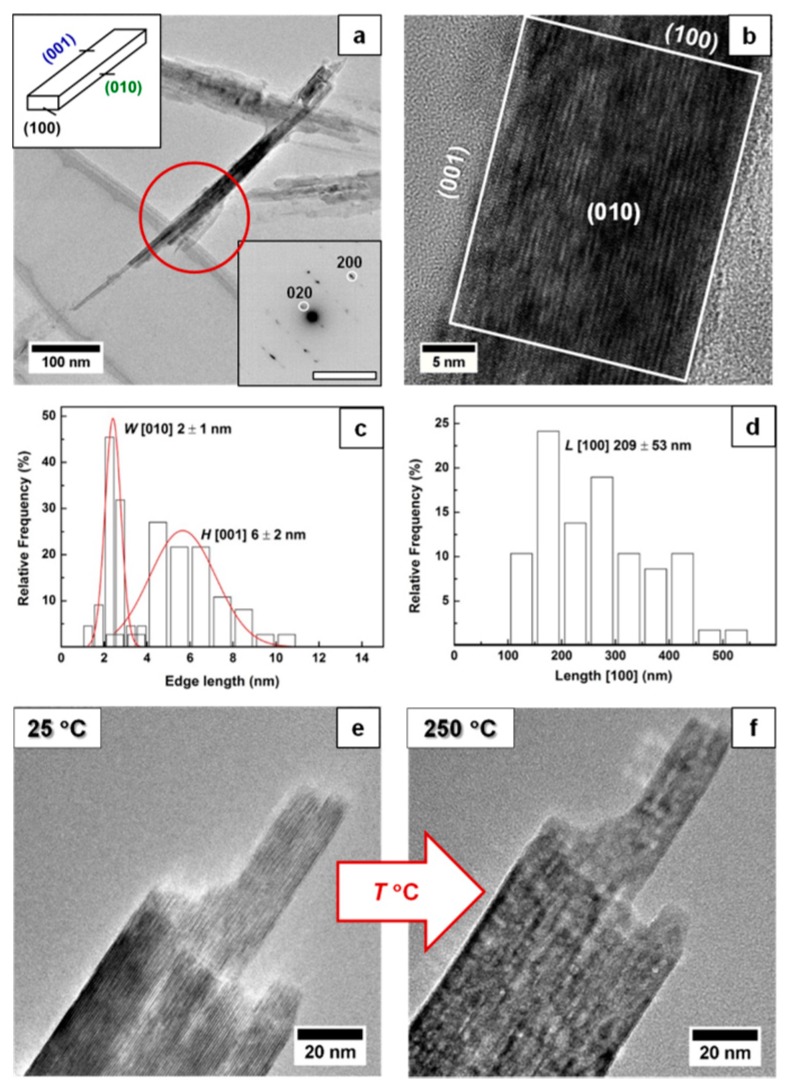
(**a**) Bright-field transmission electron microscopy (TEM) image, particle morphology (reproduced in part with permission of [39]. The Copyright Clearance Center, 2019) and selected-area electron diffraction (SAED) pattern of synthesized lepidocrocite particles. (**b**) High-resolution transmission electron microscopy (HRTEM) images of lepidocrocite nanoparticles with the corresponding termination facets. (**c**) Histogram depicting size distribution of the height and width of Lp nanoparticles. (**d**) Histogram depicting size distribution of the length of Lp nanoparticles. (**e**) Particles’ morphology before thermal transformation (25 °C). (**f**) Particles’ morphology after thermal transformation (250 °C).

Particle dimensions were measured manually from ~30 particles. The selected-area electron diffraction (SAED) pattern of each particle was taken to determine their orientation and distinguish between their height or width (see Figure 1). Pore sizes from about 200 pores were also measured manually from HAADF-STEM images. For spherical pores, the size was measured as a diameter, while for slit-shaped pores the size was measured between two edges across the slit. The analysis of the TEM images was performed using the program ImageJ [37].

Thermal Gravimetric Analysis (TGA). TGA experiments were carried out a TA Instruments Discovery TG (New Castle, DE, USA) machine in a temperature range of 30–280 °C under air, using about 5 mg of powder and Pt pans.

Adsorption Measurements. Nitrogen adsorption isotherms were measured at −196 °C using a Micromeritics ASAP 2020 (Norcross, GA, USA) porosimetry system. The samples were degassed under conditions of dynamic vacuum at temperatures of 50–280 °C for 10 h prior to measurements. Specific surface areas (S_BET_) were calculated using standard expressions for Brunauer–Emmet–Teller (BET) isotherms. For BET analyses, the uptake of nitrogen at relative pressures of *p/p_0_* = 0.05–0.18 was used. Care was taken to assure that the c-values were positive and not unphysically large. Micropore volumes were determined with the t-plot method. Total pore volume was determined from the adsorbed volume at the highest *p/p_0_* point on the isotherm. The Barrett–Joyner–Halenda (BJH) [38] adsorption model was used for the calculation of the pore volume due to presence of the artefact peak in all BJH desorption graphs.

Magnetometry. Magnetization as a function of temperature was obtained using a LakeShore vibrating sample magnetometer (Cryophysics GmbH, Darmstadt, Germany) (VSM) at the Ångström laboratory in Uppsala University.

## 3. Results and Discussion

Analysis of the material with TEM and SAED (see Figure 1) shows that the lepidocrocite nanoparticles are terminated by the (100)_Lp_, (010)_Lp_ and (001)_Lp_ faces where the width, height and length of the particle are taken along [010]_Lp_, [001]_Lp_ and [100]_Lp_, respectively. The lepidocrocite nanoparticles had a rectangular prism shape and average width *W* = 2 ± 1 nm, height *H* = 6 ± 2 nm and length *L* = 209 ± 53 nm, and an aspect ratio *AR* = *L/√*(*W·H*) ≈ 65 where many nanoparticles form agglomerates.

**Figure 2:**
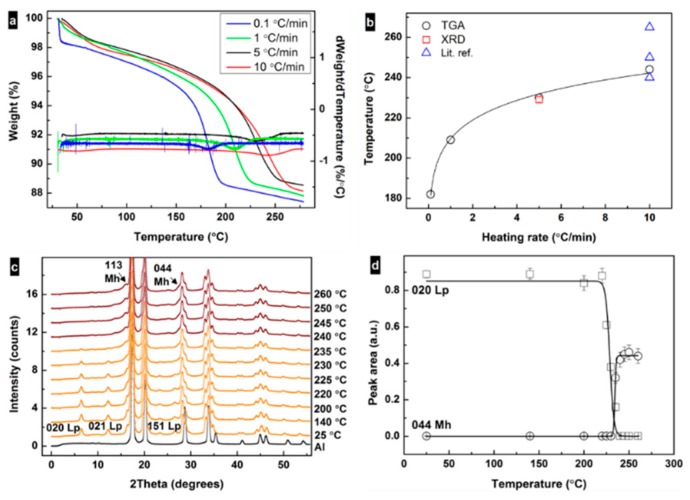
(**a**) Thermal gravimetric analysis (TGA) curves and their derivatives for the samples heated at 0.1, 1, 5 and 10 °C/min rates. (**b**) Correlation between heating rate and transformation temperature of lepidocrocite as obtained from TGA and X-ray diffraction (XRD) data and from literature [26,27,32]. (**c**) Lepidocrocite (orange) to maghemite (red) transformation upon heating at 5 °C/min under air from in situ XRD patterns. Black curve represents Al from the specimen pan. (**d**) Change in integrated intensities of the 020 lepidocrocite and 440 maghemite diffraction peaks under heating.

Figure 2a shows TGA curves for samples treated at different heating rates. The weight loss in the range ≈150–260 °C corresponds to about one molecule of water per iron oxyhydroxide (≈10.1 wt%). According to the analysis of the TGA data (Figure 2b), the transformation temperature *T*_t_^TGA^ increases from around 183 °C at the slowest rate (0.1 °C/min) to about 244 °C at 10 °C/min. The transformation temperature was also determined from the iXRD profiles using the integrated intensity of the 020_Lp_ (and 440_Mh_) peaks. Figure 2c shows iXRD patterns of a sample heated up to 280 °C at a heating rate of 5 °C/min. Under these experimental conditions, the transformation happened at 230 °C, similar to what was observed with TGA (Figure 2b). Up to ≈235 °C, XRD patterns show the presence of only a lepidocrocite phase. With the increase in temperature, the integrated peak intensity of 020_Lp_ decreases and from >230 °C the integrated peak intensity of 440_Mh_ increases (Figure 2d). Domain sizes of crystallites were calculated using the Scherrer formula with shape factor = 0.9 [40,41]. The obtained values are 5.6 ± 0.2 nm for (020)_Lp_ and 4.1 ± 0.1 nm for (440)_Mh_. The case that the domain size of (020)_Lp_ is roughly two times larger than the width of a single particle is due to the sensitivity of the XRD technique to larger particle volumes. From the analysis of the experimental data, it can be deduced that lepidocrocite–maghemite transformation happens by dehydroxylation followed by the condensation of the structure. Lepidocrocite has a layered structure with hydroxy groups located between the layers, e.g., (010) [4].

During heating, hydroxyl groups get enough energy to form a water vapor via condensation with the closest hydroxy group (H^+^ + OH^−^); the collapse of the interlayer distance can explain, e.g., the decrease in 020_Lp_ diffraction peak in the XRD pattern. The size and morphology of the nanoparticle remains the same during and after the transformation (Figure 1e), and hence the change in the surface area is likely related to the change in the particle porosity. The transformation temperature increases with the heating rate converging towards ≈250 °C (within the rates used in this study), similar to the reports in the literature [25,26,27,32] (Figure 2b). The dependence of the transformation temperature on heating rate suggests that kinetic factors dramatically influence the transformation. Indeed, the transformation has been reported to involve the following steps: (i) dehydroxylation of the Fe-OH at (020), (ii) collapse of (020) and (iii) the displacement of Fe ions to tetrahedral positions [4,5,28].

**Figure 3:**
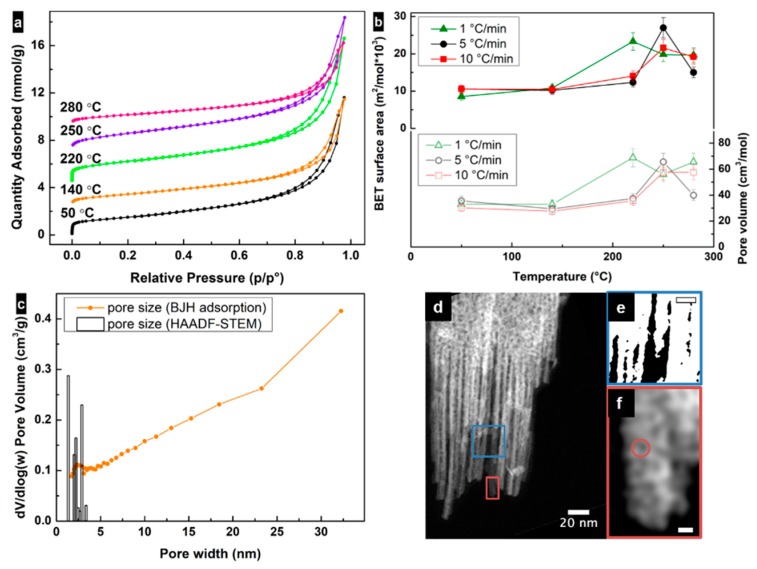
(**a**) Nitrogen (N_2_) adsorption and desorption isotherms of lepidocrocite samples treated at different temperatures with 5 °C/min heating rate. Isotherms have been offset manually along the y axis. (**b**) Correlations between samples’ surface areas (Brunauer–Emmet–Teller (BET) model) and the final temperatures of the heating (filled symbols), and between samples’ pore volume and the final temperature of the heating (hollow symbols). (**c**) Pore size distribution calculated using the Barrett–Joyner–Halenda (BJH) adsorption model and using high-angle annular dark-field imaging using scanning transmission electron microscopy (HAADF-STEM) image analysis for the sample heated up to 250 °C at 5 °C/min rate. (**d**) HAADF-STEM image, showing spherical and slit-shaped pores of the lepidocrocite sample, heated at a rate of 5 °C/min up to 250 °C. (**e**) Binary image of the magnified region in (**d**) highlighting the interparticular pores. (**f**) Enlarged image of the area shown in (**d**) highlighting small pores. Scale bars in (**e**,**f**) correspond to 10 and 2 nm, respectively.

Nitrogen surface adsorption isotherms for all samples looked similar and were assigned to type II with H3 type of hysteresis due to the presence of slit-shaped pores (Figure 3a). A trend for the change in surface area values versus the heating rate and annealing temperature was observed for all investigated samples and can be described as an increase in surface area up to the transformation temperature and then a subsequent decrease (Figure 3b). The highest surface area (≈30 × 10^3^ m^2^/mol) value was obtained for the sample treated with 5 °C/min heating rate until 250 °C.

Mesopores with a wide range of sizes were observed in all samples using the BJH adsorption model. The pore size distribution behavior was similar for all the samples (Figure 3c). The vertical raise of all isotherms at relative high pressure already indicated the presence of large mesopores related to the voids between agglomerated particles, suggesting that the largest contribution to the surface area is due to the particle size and shape. Alternatively, the pore volume for all samples increased until the transformation temperature and then decreased due to collapse of the pores (Figure 3b). Increases of 110%, 124% and 109% in pore volume were observed for samples heated up at rates of 1, 5 and 10 °C/min, respectively. A comparison of the highest pore volume values for different heating rates can be expressed as 1 > 5 > 10 °C/min. These results suggest that there are additional pores generated during the transformation, in addition to the contribution to pore volume from the particle morphology. However, microporosity (≈0.2–0.3 cm^3^/mol) was observed in all samples. The main contribution to the surface area values was then due to the particle size and shape, as well as mesopores where the contribution of micropores was small (<2 × 10^3^ m^2^/mol). The surface area values are the highest among those reported in the literature [4,25,28,30,39,42] for maghemite and lepidocrocite nanoparticles obtained from Fe^II^ and similar to other functional mesoporous materials [43,44,45,46].

The porosity of the lepidocrocite-maghemite nanoparticles was also studied at the microscopic level using HAADF-STEM. For samples heated up with 5 °C/min rate, it is possible to observe the presence of intraparticular circular and slit-shape pores with diameter/width ≈1–3 nm (see Figure 3c,d). The interparticle voids with sizes >5 nm are shown in Figure 3e. These two contributions correlate with the BJH analysis of the surface adsorption data in the small mesopore region. Line profiles drawn over the circular “pores” (see Figure 3f) show a large intensity at the pore location. This fact suggests that either the pores are closed, i.e., not open on both ends, or they are pits on the surface of the particles, i.e., open only on one side. If the pores are closed, they will not contribute to an increase in surface area or pore volume. However, if the pores are opened on one end, their formation will result in an increased surface roughness, and consequently increase the surface area. This effect will be visible at the adsorption isotherms at high partial pressures, similarly to what is seen in Figure 3a. Note that the presence of a fraction of closed pores cannot be discarded, but it is not possible to quantify from the results shown in this work.

**Figure 4:**
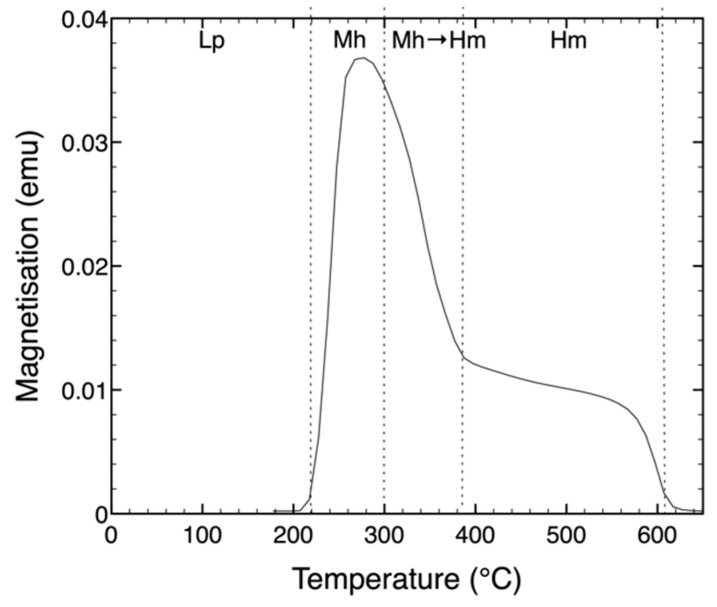
Magnetization of lepidocrocite (Lp) upon heating as a function of temperature. The sample was heated in air at 5 °C/min. Upon heating, Lp transforms into maghemite (Mh) at ≥220 °C, which subsequently transforms into hematite (Hm) upon prolonged heating at ≥ 300 °C.

The transformation of lepidocrocite was also measured using magnetometry. Figure 4 shows the changes in magnetization upon heating a lepidocrocite (Lp) sample. Upon heating at ≥220 °C, the magnetization increases dramatically with a maximum at ≈300 °C followed by a drop towards 400 °C. After a slow decay, the magnetization vanishes at ca. 600 °C. The first jump in magnetization is due to the transformation of the paramagnetic lepidocrocite (Lp) into ferrimagnetic maghemite (Mh), as has been described in the text. The following drop in magnetization is due to the transformation of ferrimagnetic maghemite into antiferromagnetic hematite, [4] which in this case shows a Curie temperature $T_{C}^{Hm}$ ≈ 600 °C.

## 4. Conclusions

In summary, the simple, reproducible and inexpensive fabrication route from Fe^2+^ to porous maghemite prismatic nanoparticles was investigated. The highest values for the surface area and porosity of these iron oxides are obtained. The porosity of maghemite nanoparticles is mainly due to the particle morphology, whereas the increase in surface roughness occurred during the transformation from lepidocrocite. The generated maghemite nanoparticles are promising materials for applications in catalysis, adsorption and energy storage. The combination of characterization techniques, such as image analysis of electron micrographs, and surface adsorption is very powerful for the understanding of porous materials.
